# A Co-Culture Model of the Developing Small Intestine Offers New Insight in the Early Immunomodulation of Enterocytes and Macrophages by *Lactobacillus spp.* through STAT1 and NF-kB p65 Translocation

**DOI:** 10.1371/journal.pone.0086297

**Published:** 2014-01-16

**Authors:** Martin Trapecar, Ales Goropevsek, Mario Gorenjak, Lidija Gradisnik, Marjan Slak Rupnik

**Affiliations:** 1 Institute of Physiology, University of Maribor, Faculty of Medicine, Maribor, Slovenia; 2 Department of laboratory diagnostics, University Clinical Center Maribor, Maribor, Slovenia; 3 University of Maribor, Faculty of Medicine, Maribor, Slovenia; Charité, Campus Benjamin Franklin, Germany

## Abstract

The early establishment of a complete microbiome has been shown to play an integral part in the development and maintenance of an intact intestine and its immune system, although much remains unknown about the specific mechanisms of immune modulation in newborns. In our study we show in a co-culture model of the undeveloped small intestine that members of *Lactobacillus* spp. influence STAT1 and NF-kB p65 nuclear translocation in both intestinal epithelial cells as well as underlying macrophages. Moreover, by using imaging flow cytometry we were able to monitor each individual cell and create a framework of the percentage of cells in which translocation occurred in challenged versus control cell populations. We also observed a significant difference in baseline translocation in intestinal cells when cultured alone versus those in a co-culture model, underpinning the importance of 3D models over monolayer set-ups in epithelial *in vitro* research. In conclusion, our work offers new insights into the potential routes by which the commensal microbiome primes the early immune system to fight pathogens, and shows how strain-specific these mechanisms really are.

## Introduction

An established microbiome is prerequisite to the early development of the intestine in newborns acquiring tolerance and immunity [1]. Growing clinical and *in vitro* evidence support the role of sensitation and priming as one of the main immunomodulatory mechanisms, by which the complete microbiome including lactic acid bacteria (LAB) act beneficially in terms of increasing the immune status of the host [2]–[5]. The role of priming is to potentiate early responses and to raise the level of alertness of mononuclear cells against pathogens without actual activation of the cells. In this process bacteria-derived extracellular stimuli such as lipopolysaccharides and lipoteichoic acids induce TLR-dependent activation of NF-kB signaling as well as secretion of type I interferons, extended to the activation of STAT1, in eneterocytes, followed by a translational cascade reaching macrophages and other cells of the innate immune system [6]–[8]. A dysregulation in those signaling cascades in early intestinal developmental often results in necrotizing enterocolitis (NEC), an inflammatory bowel necrosis of premature infants [9]. Interestingly, NEC in newborns is marked with a high infiltrate of macrophage leukocytes in affected areas and a very low count of lymphocytes, which underpins macrophages as the first line of defense, especially in the premature gut [10].

While the interaction between commensals and the adult gastro-intestinal tract is fairly well understood, many questions remain unanswered on the acquisition of intestinal immunity during first months after birth.

In our previous publications we have shown that members of *Lactobacillus plantarum*, strains PCS 20 and PCS 26 activate the production of reactive oxygen species, IL-6 and IFN-γ in undeveloped intestinal epithelial cells (IEC) as well as monocytes, orchestrating an increased anti-viral response against rotavirus, transmissible gastroenteritis virus and vesicular stomatitis virus [11]–[14].

In conjunction with the findings of other authors that have shown individual *lactobacillus* strains to induce STAT1 and NF-kB shifts in adult IEC, we raise several questions and concerns that have yet to be elucidated. Current available data on the interaction between LAB, IEC and gut associated lymphoid tissue (GALT) is mostly based on average gene expression profiles and quantification of inflammatory products. Therefore, the question remains: what happens on the level of each individual cell and on its proteome level? In addition, virtually nothing is known about how many cells from a given population of IEC and GALT actually engage in an immunomodulatory mechanism when challenged with LAB. Further, prior studies in this direction have been performed on transformed or cancer derived cell lines like CaCo-2 and HT-29, which are known to differ from a healthy *in vivo* environment due to their difference in glycosylation and phenotype [15], [16]. Finally, the majority of studies were designed as monolayer models with the lack of associated cell types despite the need of IEC for intracellular feedback.

**Figure 1:**
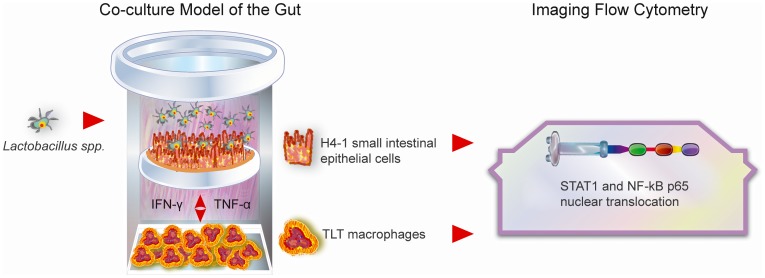
Graphic outline of the study. By using imaging multicolor flow cytometry we have monitored the translocation of NF-kB p65 and STAT1 in untransformed polarized human neonatal small intestinal epithelial cells H4-1, challenged with different *Lactobacillus spp.* strains, and in macrophage TLT cells that have been simultaneously co-cultured in a reductionist human 3D model of the immature gut.

The aim of our work was to shed more light on the mechanics of immunomodulation by specific commensal bacteria in the developing intestine and at the same time to present a reliable alternative model for gut immunology studies. By using imaging multicolor flow cytometry we have monitored the translocation of NF-kB p65 and STAT1, two of the most important intracellular orchestrators of an antimicrobial response, in untransformed polarized human neonatal small intestinal epithelia, challenged with different *Lactobacillus spp.* strains, and in macrophage cells that have been simultaneously co-cultured in a reductionist human 3D model of the immature gut (**Figure 1**). Imaging multicolor flow cytometry allowed us not only to monitor each individual cell but also to create a picture of how many cells from a certain population initiated cytoplasmic shifts [17]. Additionally, we show that not only cell culture selection is important in this type of research but also the culturing technique itself. Epithelia act differently when grown on plastic surfaces than when grown on microporous membranes and need the presence of other associated cell types like macrophages and dendritic cells to show *in vivo*-like characteristics.

## Methods

### Bacteria

Cultures of *Lactobacillus plantarum* strains PCS 20, PCS 26 (Bionutritech, France) and *Lactobacillus rhamnosus* LGG (ATCC, UK) were maintained at − 80°C in 20% (v/v) glycerol (Merck, Darmstadt, Germany) with MRS broth (Merck). Propagation, prior testing on cell lines, was later done in MRS broth (Merck, Darmstadt, Germany) for 24 h at 37°C and under anaerobic conditions by the use of Anaerogen (Oxoid Ltd, Hampshire, UK).

### Cell lines

The following cell lines were used: H4-1 (non-transformed human neonatal small intestinal cells) and TLT (non-transformed human monocyte/macrophages). H4-1 and TLT were derived from healthy tissues [10], [16].

Cells were generally maintained in Dulbecco's modified Eagle's medium (DMEM) supplemented with 10% fetal calf serum, L-glutamine 2 mmol/l, penicillin (100 U/ml, Sigma) and streptomycin (1 mg/ml) at 37°C in 5% CO_2_ atmosphere in tissue culture flasks.

### Co-culture model of the neonatal gut

We cultured H4-1 cells on 12 well Transwell plates with 22 µm microporous inserts (Sigma-Aldrich, UK). The size of the pores did not allow migration of bacteria into the basal compartment. Simultaneously TLT monocytes were cultured on separate 12 well plates without inserts.

Once epithelia reached a constant trans-epithelial resistance above 800 Ohms and monocytes were confluent, we moved the inserts with H4-1 cells to the plate with TLT cells thereby creating a co-culture 3D model. After the model was established, epithelial cells were exposed for 24 hours to 1×10^7^ CFU/ml of each strain separately, as well as 100 ng ml^−1^ of IFN-gamma (Sigma-Aldrich, UK) and 10 ng ml^−1^ of TNF-alpha (Sigma-Aldrich, UK) for a positive control. We took for the negative control co-culture cells that were not challenged. In the same time we grew H4-1 cells on inserts without the presence of TLT in the basal compartments and TLT alone without H4-1 in order to determine the influence of co-culture on translocation. After a 24 incubation period we harvested the cells by trypsinization and prepared them for imaging multicolor flow cytometry.

### STAT1 and NF-kB translocation

Cells were trypsinized, washed with PBS and centrifuged to obtain a pellet of about 10^6^ cells in 100 µL in polypropylene tubes. Cells were stained with the following antibodies according to the manufacturers protocols: anti-NF-kB p65 rabbit polyclonal (Santa Cruz Biotechnology, Germany), goat anti-rabbit Cy3 (Santa Cruz Biotechnology, Germany), Alexa Fluor 647 mouse anti-STAT1 (BD Biosciences, UK) and 7AAD (BD Biosciences, UK) for staining of the nucleus. Cell images were acquired using the ImageStreamX multispectral imaging flow cytometer (Amnis Corporation, Seattle, USA), collecting 5000 events per sample at 40× magnification. A 488 nm wavelength laser was used to excite Cy3-labeled NF-kB p65 as well as 7AAD and a 658 nm laser for Alexa Fluor 647-labeled STAT1. The fluorescence images were acquired using the 560−595 nm, 595-660 nm and 660−745 nm spectral detection channels, respectively. For the triple-stained cells 3 single-stained controls were used to compensate fluorescence between channel images on a pixel-by-pixel basis. Cell images were analyzed using IDEAS image-analysis software (Amnis Corporation).

Gating on bivariate plot of aspect ratio versus cell area was first used to isolate a population of single cells. Cells within the focal plane were further selected using a two-dimensional plot of image contrast versus root-mean-squared (rms) gradient. The software compared the location of probes (NF-kB, STAT1) with the location of the nucleus in each acquired cell, running a probe similarity algorithm. The subpopulation of cells in which a translocation occurred was calculated and expressed in percentage. Further samples were compared to the control without bacterial strains.

### Statistics

Each experiment was performed in triplicate and results are expressed as mean percentages of each analyzed subset of cells in which nuclear translocation has occurred. Numbers and percentages of cells positive for translocation were calculated with the IDEAS image-analysis software (Amnis Corporation, Seattle, USA). Since the occurrence of translocation are yes/no events they are considered descriptive variables and the χ^2^ test was used to evaluate the statistical significance of the differences between groups. We used SPSS 18.0 (SPSS Inc., Chicago, USA) to calculate the χ^2^ tests and marked with an asterisk (“*”) values significantly different from the control (p<0.05).

## Results

### *Lactobacillus spp.* trigger STAT1 and NF-kB p65 translocation in H4-1 small intestinal epithelial cells cultured in a human 3D model of the gut

**Figure 2:**
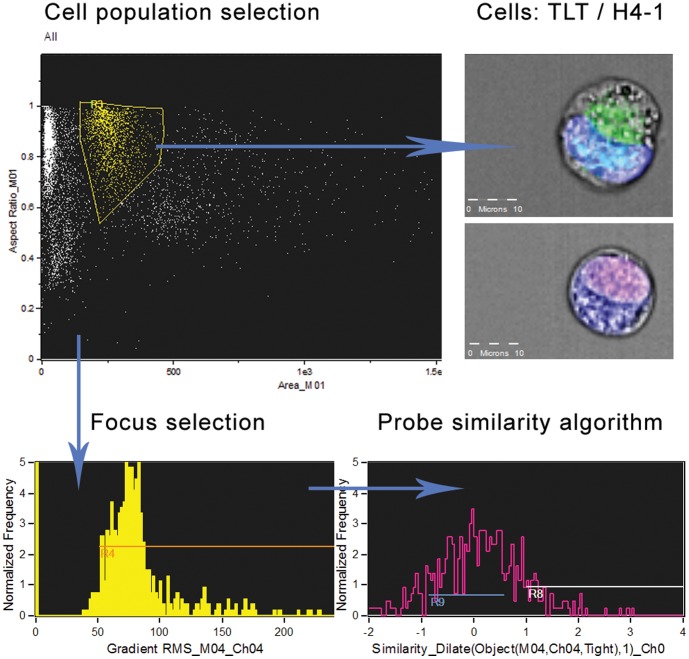
Analysis of cell populations with imaging flow cytometry. Cells were stained with Cy3-labeled NF-kB p65 as well as 7AAD and Alexa Fluor 647-labeled STAT1. Images were acquired using the ImageStreamX multispectral imaging flow cytometer, collecting 5000 events per sample at 40× magnification and analyzed using IDEAS image-analysis software. Gating on bivariate plot of aspect ratio versus cell area was first used to isolate a population of single cells. Cells within the focal plane were further selected using a two-dimensional plot of image contrast versus root-mean-squared (rms) gradient. The software compared the location of probes (NF-kB, STAT1) with the location of the nucleus in each acquired cell, running a probe similarity algorithm.

**Figure 3:**
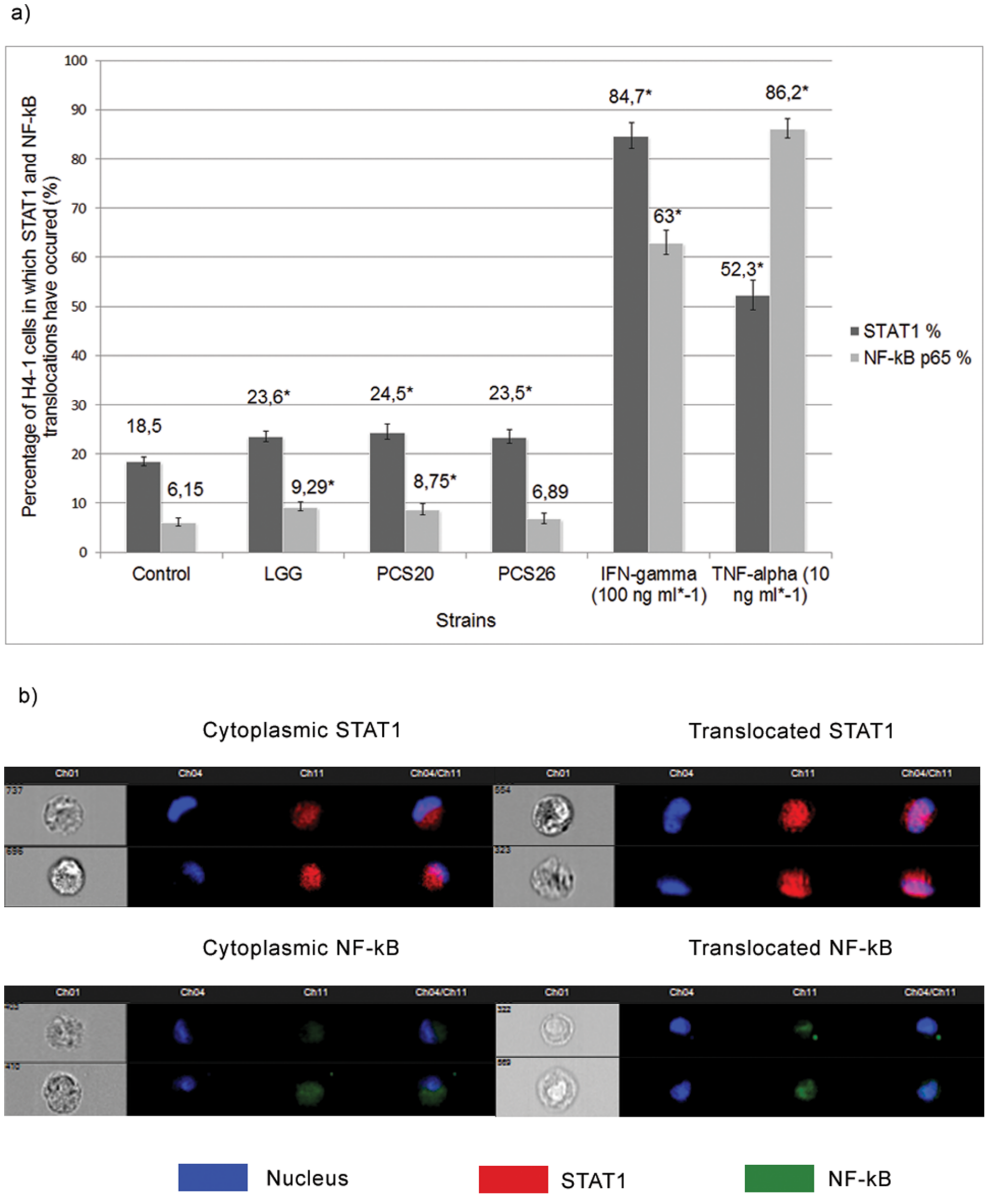
NF-kB p65 and STAT1 translocations in small intestinal epithelial cells. a) Percentage of H4-1 small intestinal epithelial cells in co-culture with TLT macrophages in which translocation of STAT1 and NF-kB occurred after treatment with different bacterial strains and positive controls. b) Acquired images of H4-1 cells with cytoplasmic or translocating probes. * indicates significant differences from control cells (p<0.05).

Untransformed H4-1 IEC co-cultured with TLT macrophages on microporous membranes and treated with different bacterial strains were stained with antibodies against STAT1 as well as NF-kB p65 and further analyzed with imaging flow cytometry (**Figure 2** **)**. In 18.5% of cells a STAT1 translocation from the cytoplasm to the nucleus was observed when cultured without bacterial strains. Addition of *Lactobacillus spp.* increased the number of cells in which nuclearization of STAT1 occurred as shown in **Figure 3a,b** although no significant differences could be detected among individual strains LGG, PCS 20 and PCS 26.

NF-kB p65 translocation was observed in 6.15% of unchallenged cells. Addition of LGG and PCS 20 strains increased translocation to 9.29% and 8.75%, respectively. PCS 26 did not statistically significantly influence p65 translocation (**Figure 3a,b**).

IFN-γ and TNF-α served as positive controls, both significantly increasing the number of cells in which STAT1 and NF-kB shifts have occurred.

### Nuclearization of STAT1 and NF-kB in epithelia consequently induces translocation in basolateral TLT macrophages

Considerable differences were observed in regard to STAT1 and p65 translocation in basolateral TLT macrophages co-cultured in the basal compartments of the 3D model of the gut during bacterial challenge.

Translocation of STAT1 was observed in 16.5% of the control TLT cells cultured without bacteria present.

**Figure 4:**
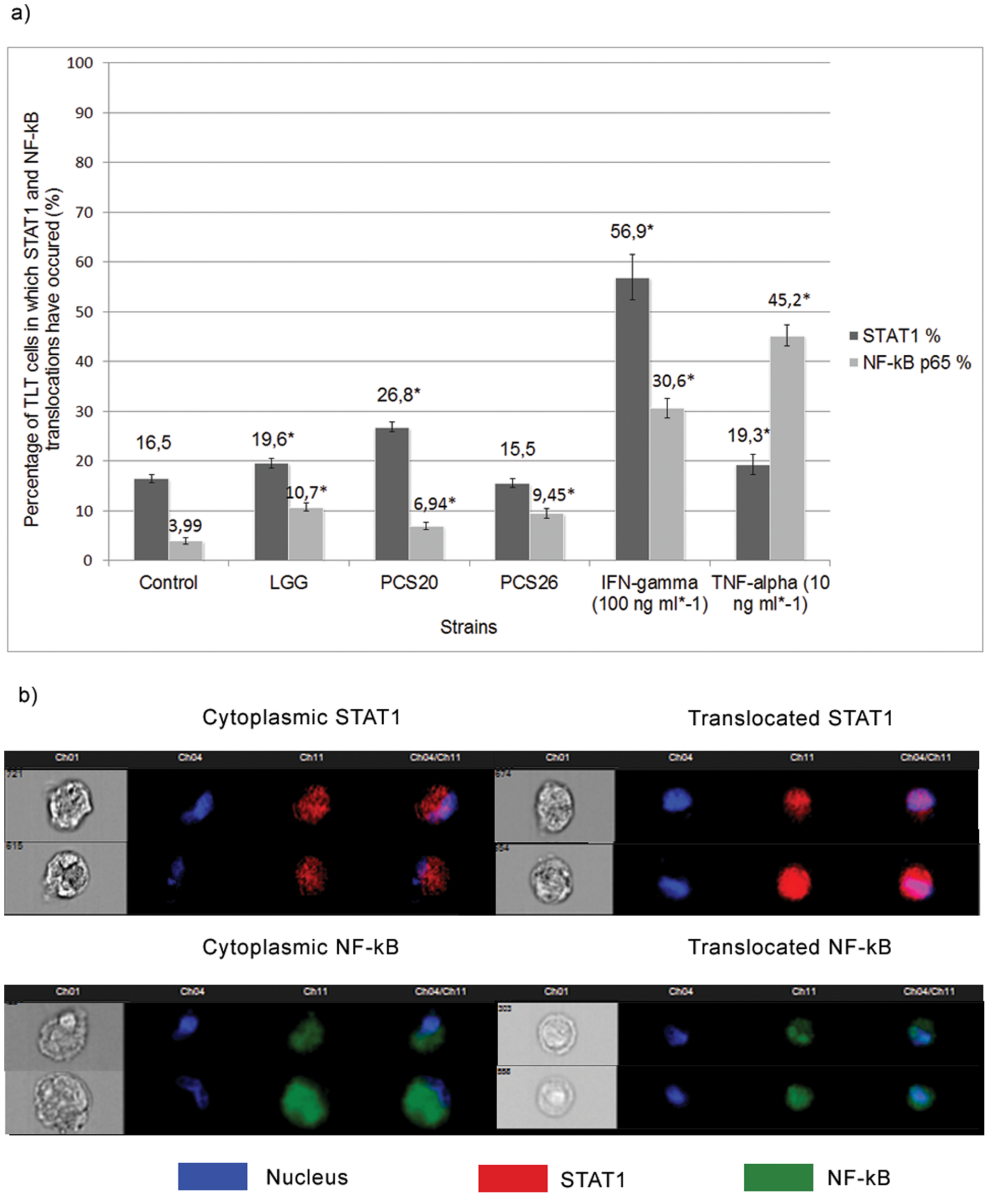
NF-kB p65 and STAT1 translocations in TLT macrophages. a) Percentage of TLT macrophage cells in basolateral co-culture with H4-1 cells in which translocation of STAT1 and NF-kB occurred after treatment with different bacterial strains and positive controls. b) Acquired images of H4-1 cells with cytoplasmic or translocating probes. * indicates significant differences from control cells (p<0.05).

Exposure of cells to LGG and PCS 20 cells increased STAT1 nuclearization to 26.8% and 19.6%, respectively. PCS 26 did not significantly increase STAT1 translocation. (**Figure 4a,b**).

4% of TLT cells showed a translocation of NF-kB p65 in the control model whereas the challenge with LGG increased translocation to 10.7%, PCS 20 to 6.9% and PCS 26 to 9.5% (**Figure 4a,b**).

A considerably higher number of cells treated with IFN-γ and TNF-α were positive for STAT1 and NF-kB translocations.

### Epithelial cells behave differently in regard to STAT1 and NF-kB translocation when cultured alone or in co-culture

**Figure 5:**
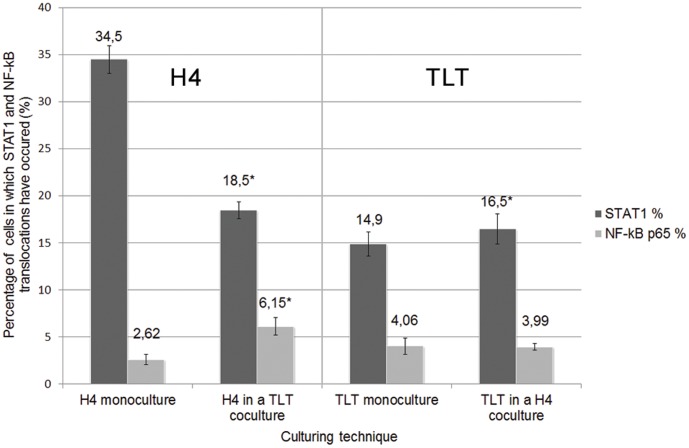
Baseline translocations in regard to the culturing technique. Percentages of untreated H4-1 and TLT cells in which baseline STAT1 and NF-kB cytoplasmic shifts have been observed in regard to the culturing technique. * indicates significant differences between models (p<0.05).

When H4-1 IEC were cultured alone on inserts without the presence of macrophages, 34.5% of the cell population showed STAT1 translocation and 2.62% showed nuclearization of NF-kB p65. In the H4-1 and TLT co-culture the percentage of H4-1 cells with translocating STAT1 decreased to 18.5% and the percentage of cells translocating p65 increased to 6.15% (**Figure 5**).

Among TLT cells, co-culturing increased the percentage of STAT1 translocation among TLT cells from 4% to 16.5%. However, translocation of NF-kB did not significantly differ between the co-culture and the monolayer models.

## Discussion

The development of a normal and functioning immune system is largely dependent on the interaction of the newborn with the commensal microbiome which is one of the key players assuring intact immune homeostasis [18]. This constant interplay between microorganisms, the gut, and associated lymphoid tissue is believed to result in a persistent stimulation, sensitation, and priming of the hosts immune system [6]. We have shown that specific strains of *Lactobacillus* are able to trigger increased translocation of STAT1 and NF-kB p65 in untransformed intestinal epithelial cells and that this signal translates further into macrophages. NF-kB is a nuclear factor composed of several protein subunits regulating DNA transcription that is present in its inactive form in the cytoplasm, bound to the inhibitor molecule, IkB, while under non-stimulatory conditions. After pro-inflammatory stimuli trigger signaling pathways, IkB is phosphorylated by IKK, targeting it for ubiquitination by E3-SCFβ-TrCP. Once freed from IkB, NF-kB subunit p65 (RelA) migrates into the nucleus, where it binds to target promoters and activates transcription of effector genes, functioning as a release initiator of pro-inflammatory cytokines, like TNF-α, IL-8 and others [6], [18]-[20]. Although other NF-kB subunits exist, only p65 is needed to promote IL-8 [21].

While translocation of p65 may result in various scenarios, translocation of STAT1 is triggered by interferon family proteins that further act as signaling molecules for an anti-microbial response [7], [22]. Following tyrosine phosphorylation of STAT1 by receptor associated Janus kinases, STAT1 translocates into the nucleus where it binds to a regulatory DNA element termed gamma activated sequence. Recent data have shown that TLR-9 signaling mediates the anti-inflammatory effects of probiotics. Systemic administration of TLR-9 ligands reduces the severity of colonic injury and inflammation in models of experimental colitis [23], in part through the TLR9-induced production of type I IFNs.

De Kivit et al. have shown that increase in IFN-γ secretion via TLR9 ligands also depended on the presence of IEC [24]. These results are in line with our previous findings postulating that those strains induce the production of IFN- γ resulting also in an increased anti-viral response.

Not all cells were influenced by bacterial challenge: approximately 20% for STAT1 and 10% for NF-kB p65 translocation. Considerable differences could be observed while comparing translocations in IEC and macrophages. PCS 20-challenged IEC showed the highest degree of STAT1 translocation, and the effect was even greater in macrophages.

The strain PCS 26, in contrast, did not increase the translocation rate of p65 in IEC but influenced a significantly higher rate in underlying macrophages; however, it did not statistically alter translocation of STAT1 in either cell type. Similarly *Lactobacillus acidophilus* has been found to increase TNF-α mRNA in leucocytes co-cultured with CaCo-2 cells, whereby CaCo-2 cells remained hypo responsive [19].

LGG showed the highest overall influence on p65 translocation. This finding aligns with previous reports by Sanz et al. that *L. plantarum* BFE 1685 and LGG promoted increased TLR-2 receptor and IL-8 expression in macrophages [4] and by Miettinen et al. that LGG triggers STAT and NF-kB DNA binding in primary monocytes [25].

Moreover *Lactobacillus plantarum* and LGG have been shown to induce inflammatory cytokines also in dendritic cells, driving Th1 T cell proliferation in response to *Salmonella,* with *L. plantarum* similarly being the most potent inducer [26].

Several clinical studies that have shown specific lactic acid strains to promote sensitation and Th1-type immunity support our *in vitro* findings [5], [27] however future studies should be conducted to show whether this effect can be considered systemic.

Our data and prior observations, that several other *Lactobacillus* strains have been shown to induce anti-inflammatory responses [28], demonstrate that the observed effects are very strain specific [5], [18]. This difference has important implications for, among other things, probiotic supplementation as not every strain is suitable for every consumer or suitable at all, as in the case of immunocompromised newborns.

We have further shown that the baseline level of STAT1 translocation in non-transformed IEC cultured alone or in the presence of macrophages differs drastically, whereas macrophages alone did not seem to be as dependent on co-cultivation. We speculate that the epithelium requires macrophage-derived soluble factors and co-stimulatory surface molecules as feedback regulators that “calibrate” epithelial immune function. Haller et al. have shown in a CaCo-2/leucocyte model how a challenge with non-pathogenic bacteria results in a pro-inflammatory response that is later feedback-controlled by T cells via IL-10 and STAT3 that prevents inflammatory escalation [19], a model also proposed by Hu et al. [29] and Parlesak et al. [30]. This underpins the importance of co-culture or 3D models for studies of host-microbe interactions and puts in question earlier immunomodulatory studies done in IEC monolayer set-ups, a concern raised previously [16], [18]. Variances in model and cell line selection may partially explain apparently contradictory data found in literature on the pro- or anti-inflammatory actions of most common industrial strains such as *Lactobacillus rhamnosus* GG.

## Conclusion

We conclude that members of *Lactobacillus spp.* have the ability to immunomodulate macrophages as well as immature enterocytes in the developing intestine via NF-kB p65 and STAT1 translocation thereby driving enhanced antimicrobial alertness in newborns which in turn offers greater protection against pathogens during the development of acquired immunity. Future clinical studies should aim to elucidate whether probiotic interventions in infants might contribute to more balanced p65 and STAT signaling development in the immature gut as well as the gut-associated immune system.
